# PLD1 is overexpressed in an ER-negative MCF-7 cell line variant and a subset of phospho-Akt-negative breast carcinomas

**DOI:** 10.1038/sj.bjc.6603926

**Published:** 2007-08-28

**Authors:** J M Gozgit, B T Pentecost, S A Marconi, R S J Ricketts-Loriaux, C N Otis, K F Arcaro

**Affiliations:** 1 Department of Veterinary and Animal Sciences, University of Massachusetts, 639 North Pleasant Street, Morrill 1 North, Amherst, MA 01003-9298, USA; 2 Wadsworth Center, New York State Department of Health, Albany, NY 12202, USA; 3 Department of Pathology, Baystate Medical Center, Springfield, MA 01199, USA

**Keywords:** PLD1, Akt, breast cancer, rapamycin, TMX2-28 cells

## Abstract

We have used a novel variant of the human oestrogen receptor (ER)-positive MCF-7 cell line, TMX2-28, as a model to study breast cancer. TMX2-28 cells show no detectable levels of mRNA or protein expression for the ER and express basal cytokeratins (CKs) 5, 14, and 17. cDNA microarray comparison between TMX2-28 and its parent cell line, MCF-7, identified 1402 differentially expressed transcripts, one of which was, phospholipase D1 (PLD1). Using real-time RT–PCR, we confirmed that PLD1 mRNA levels are 10-fold higher in TMX2-28 cells than in MCF-7 cells. We next examined PLD1 expression in human breast carcinomas. Phospholipase D1 mRNA levels were higher in breast tumours that expressed high-mRNA levels of basal CKs 5 and/or 17, but PLD1 mRNA levels were not significantly higher in ER-negative tumours. Phospholipase D1 protein was overexpressed in 10 of 42 (24%) breast tumours examined by IHC. Phospholipase D1 was overexpressed in 6 of 31 ER-positive tumours and 4 of 11 ER-negative tumours. Phospholipase D1 was overexpressed in three of the four tumours that showed high CK5/17 expression. Five PLD1-positive tumours were negative for phospho-Akt expression, but positive for phospho-mammalian target of rapamycin (mTOR) expression. The other five PLD1-positive breast tumours showed positive expression for phospho-Akt; however, only two of these cases were positive for phospho-mTOR. In this study, we report that PLD1 and phospho-mTOR are coexpressed in a subset of phospho-Akt-negative breast carcinomas.

Phospholipase D1 (PLD1) is a phospholipid-metabolising enzyme that catalyses the hydrolysis of phosphatidylcholine to phosphatidic acid (PA) and choline, consequently activating a second messenger signalling cascade consisting primarily of PA (Foster and Xu, 2003). Phospholipase D1 is activated by upstream mitogens and is dependent on the inositol phospholipid, phosphatidylinositol (PI)-4,5-bis-phosphate (PIP2), for its complete activation. Phospholipase D1 is involved in various cellular functions, including vesicle trafficking, cell proliferation, and cell-cycle regulation (Foster and Xu, 2003). Elevated PLD1 activity is associated with cells that have high-tyrosine kinase activity (Joseph *et al*, 2001), and increased PLD1 activity has been shown to generate survival signals, causing cells to evade apoptosis (Zhong *et al*, 2003).

The mammalian target of rapamycin (mTOR) is a protein kinase that controls the phosphorylation activity of several downstream effectors and it is generally thought that the serine/threonine kinase, Akt, is the primary driver of mTOR activation (Schmelzle and Hall, 2000; Carraway and Hidalgo, 2004; Thomas, 2006). Additionally, in response to mitogenic signals, the second messenger PA binds to and activates mTOR by interacting with the domain of mTOR that is also targeted by rapamycin (Fang *et al*, 2001; Chen and Fang, 2002). It has been reported that PA is sufficient to activate downstream mTOR effectors, and that PLD1 activates the PA/mTOR pathway controlling cell size and growth (Fang *et al*, 2003). Mammalian target of rapamycin, in its role of regulating protein translation and controlling cell growth, has been implicated in cancer progression (Schmelzle and Hall, 2000; Ruggero and Pandolfi, 2003), and several analogues of rapamycin, a natural mTOR antagonist, are in clinical trials for various cancers, including advanced and oestrogen receptor (ER)-dependent breast cancer (Carraway and Hidalgo, 2004).

ER expression level in tumours has been the most important clinical diagnostic parameter for breast cancers for the past 30 years (Osborne, 1998). In recent years, several researchers have used cDNA microarray technology to identify a molecular portrait or gene expression signature of breast cancers that correlates with clinical outcome (Perou *et al*, 2000; Sorlie *et al*, 2001; Abd El-Rehim *et al*, 2004). These studies have shown that breast tumours can be subgrouped based on their cytokeratin (CK) expression profile. Luminal-like breast tumours express (CKs) 8 and 18, whereas basal-like tumours express CKs 5, 17 and/or 14 and are often associated with a poor clinical outcome for the patient. We used an ER-negative variant of the MCF-7 cell line, TMX2-28 (Fasco *et al*, 2003), which shows characteristics relevant to basal-like tumours, as a model to study this aggressive subset of breast cancers. TMX2-28 cells maintain a similar epithelial morphology to MCF-7 cells and have not undergone epithelial–mesenchymal transition. However, TMX2-28 cells are highly invasive compared to MCF-7 cells as determined by their ability to invade through a Matrigel layer (Gozgit *et al*, 2006). cDNA microarray comparison of MCF-7 cells and TMX2-28 cells identified the overexpression of PLD1 in the TMX2-28 cell line. Recently, Chen *et al* (2005) showed that PLD1 and phospho-Akt are inversely expressed in a small subset of breast cancer cell lines. The authors reported that MDA-MB-231 cells have high-PLD1 activity, which is responsible for promoting mTOR-dependent survival signals in these breast cancer cells that do not express active PI3K/Akt survival signals. Likewise, MDA-MB-435S cells have low levels of PLD1 activity and are dependent on PI3K/Akt survival signals. The results from Chen and colleagues are intriguing and in this study, we have used clinical tissues to evaluate the expression of PLD1 with phospho-Akt and phospho-mTOR in breast carcinomas to help facilitate patient selection for mTOR-targeted therapies. We show that PLD1 is expressed in a subset of phospho-Akt-negative tumours that maintain phospho-mTOR expression.

## MATERIALS AND METHODS

### Cell culture

TMX2-28 cells were kindly provided by Dr John Gierthy (Wadsworth Center). MCF-7 cells were purchased from the American Type Culture Collection (ATCC), and 184, 184A1, and 184AA2 cell lines were a generous gift from Dr Martha Stampfer (Ernest Orlando Lawrence Berkeley National Laboratory). Cell cultures were maintained in complete growth medium and subcultured according to the appropriate manufacturer's/supplier's protocols. TMX2-28 and MCF-7 cells were grown in Dulbecco's modified Eagle's medium (without phenol red) supplemented with 5% cosmic calf serum (Hyclone, Logan, VT, USA), 2.0 mM L-glutamine, 0.1 mM nonessential amino acids, 10 ng ml^−1^ insulin, 100 U ml^−1^ penicillin, and 100 *μ*g ml^−1^ streptomycin (referred to as DC_5_). Cell lines 184, 184A1, and 184AA2 were cultured according to Dr Martha Stampfer's protocol as posted on her website (http://www.lbl.gov/LBL-Programs/mrgs). Cell cultures were maintained in a 37°C humidified incubator with either 5% CO_2_ for TMX2-28 and MCF-7 cells or 2% CO_2_ for all 184 cells.

### Human tissue specimens

All tissue specimens (frozen-breast tumours, *n*=30; formalin-fixed, paraffin-embedded (FFPE) breast tumours, *n*=46; and FFPE tissues from reduction mammoplasty, *n*=10) were retrieved from Baystate Medical Center, Department of Surgical Pathology, and were identified numerically, maintaining patient anonymity. Clinical pathology reports accompanied all cases, providing data on tumour type, histological grade, tumour size, stage, hormone receptor, and HER2 expression status. IRB approval for this study was obtained from Baystate Medical Center. All 76 breast tumour samples were from primary breast carcinomas.

### RNA purification

RNA was isolated from cell cultures during exponential growth phase (70–80% confluence). Cells were washed two times with PBS, and RNA was isolated with TRI Reagent (Molecular Research, Cincinatti, OH, USA) according to the manufacturer's protocol. Isolated RNA was further purified using Qiagen RNeasy kit with on-column DNase digestion (Qiagen, Valencia, CA, USA). Tissue from frozen-breast carcinomas was sectioned to be not thicker than 0.5 cm, and immediately placed in prechilled RNA Later-Ice (Ambion, Austin, TX, USA) and incubated until processing 24 h later. Tissue samples were weighed, and then sectioned with a scalpel, and 20–30 mg were homogenised using the Tissue-Tearor (BioSpec Products, Bartlesville, OK, USA). RNA was isolated using the Qiagen RNeasy Fibrous Tissue kit (Qiagen), which includes a 10 min proteinase K digestion following homogenisation. All RNA samples were quantified by measuring the absorbance at 260 nm with the GeneQuant (Amersham Biosciences, Piscataway, NJ, USA), and RNA quality was assessed by gel electrophoresis.

### cDNA microarray

To compare TMX2-28 to MCF-7 cells at the genomic level, we performed global gene-expression analysis by cDNA microarray technology. TMX2-28 and MCF-7 cells were grown for 48 h in charcoal/dextran-treated fetal bovine serum (FBS, Hyclone), and RNA was isolated as described above when cells reached 80% confluence. The integrity of the RNA was evaluated by gel electrophoresis and sent to the Affymetrix Genechip Resource in the Keck Biotechnology Resource Laboratory at Yale University (New Haven, CT, USA). RNA was then tested with the Agilent 2100 Bioanalyzer, and global gene expression was evaluated on the Affymetrix Human Genome U133 Plus 2.0 Array, consisting of over 47 000 transcripts, according to the Affymetrix Genechip Resource standard operating procedures.

### Real-time RT–PCR

Real-time RT–PCR was performed as described previously (Gozgit *et al*, 2004). RNA (75 ng) samples were reverse transcribed and amplified using the One-Step RT–PCR kit (Qiagen) in the Roche Light Cycler (Roche Applied Sciences, Indianapolis, IN, USA). Generation of amplified products was monitored over 45 PCR cycles by fluorescence of intercalating SYBR Green. Gene-specific primers and 10-fold dilutions of RNA were used to create a standard curve for each gene, which was stored for later quantification of relative mRNA levels with the Roche Light Cycler software. The mRNA levels were normalised to mRNA levels of hypoxanthine ribosyltransferase (HPRT) as described previously (de Kok *et al*, 2005).

The following gene-specific primers were designed using Primer3 (Rozen and Skaletsky, 2000): PLD1 NM_002662 AATCGTTGGAGGTTGGACTG (sense, nt 1220), AGACGGTGGAT GACACATGA (antisense, nt 1406); CK5 NM_ 000424 CAACCCACTAGTGCCTGGTT (sense, nt 41) ATAGCCACCCACTCCAC AAG (antisense, nt 267); CK14 NM_000526 TTCTGAACGAGA TGCGTGAC (sense, nt 909), GCAGCTCAATCTCCAGGTTC (antisense, nt 1097); CK17 NM_000422 GCTGCTACAGCTTTGGCTCT (sense, nt 224) TCACCTCCAGCTCAGTGTTG (antisense, nt 341); CK8 NM_002273 TGAGGTCAAGGCACAGTACG (sense, nt 847), TGATGTTCCGGTTCATCTCA (antisense, nt 1007); CK18 NM_000224 CACAGTCTGCTGAGGTTGGA (sense, nt 892), GAGCTGCTCCATCTGTAGGG (antisense, nt 1055) CK19 NM_002276 TGAGTGACATGCGAAGCCAATAT (sense, nt 799), GCGACCTCCCGGTTCAAT (antisense, nt 901); CK20 NM_002276 TCCCAGAGCCTTGAGATAGAACTC, GTTGGCTAACTGGCTGCT GTAAC (received from Dr Fasco, Wadsworth Center); HPRT NM_000194 ACCCCACGAAGTGTTGGATA (nt 587, sense), AAGCAGATGGCCACAGAACT (nt 834, antisense); and ER*α* (Fasco *et al*, 2003).

### Tissue microarray and immunohistochemistry

Two tissue microarrays (TMAs) were designed with 46 FFPE breast carcinomas in which haemotoxylin and eosin-stained sections were used to identify the infiltrating carcinoma cells. Three 0.8 mm diameter cores were extracted from each donor block and re-embedded into a recipient paraffin-block containing holes spaced 0.8 mm apart. Four-micrometre sections were placed on charged slides, deparaffinised in xylene, and re-hydrated in graded ethanols. Slides were rinsed with H_2_O and incubated in Citra Plus Buffer (BioGenex, San Ramon, CA, USA) under the following conditions for antigen retrieval: microwaved for 3 min, cool 1 min, heated at 98°C for 10 min, and cooled for 20 min. Immunohistochemistry (IHC) was performed on the DakoAutostainer (Carpinteria, CA, USA) using Dako Envision Plus labelled polymer-HRP reagents. Negative controls (no primary antibody) were included for each tissue section. Primary antibodies were obtained from Santa Cruz Biotechnology (Santa Cruz, CA, USA): PLD1 (1 : 20, clone F-12); Dako (Carpinteria, CA, USA): CK5 (1 : 50, clone D5/16 B4) and CK17 (1 : 25, clone E3); Cell Signaling Technology (Danvers, MA, USA): phospho-Akt (1 : 25, no. 9277 and no. 3787, phospho-mTOR (1 : 25, no. 2971), and phospho-p70S6K (1 : 100, 9277). Scoring for the two antibodies for phospho-Akt showed ∼90% consistency; however, some tumours were scored positive when only one antibody showed staining. Immunoreactivity was scored by a pathologist (CN Otis) at two separate times, and intravariability observation was <10%. Immunohistochemistry sections were examined for overall staining intensity and assigned a numerical score, where 0=negative staining, 1=weak or basal level, 2=moderate, and 3=strong staining. A score of moderate or strong expression is considered overexpressed. Owing to inadequate tissue samples from four tumours represented on the TMA, PLD1 expression was evaluated in only 42 of the 46 tumours; therefore, all other IHC analyses were limited to these 42 cases. Immunohistochemistry analysis of tissue specimens from reductive mammoplasty was performed on individual tissue sections.

### Western immunoblotting

Cell lysates were collected in SDS buffer (1% SDS, 0.06 M. Tris–HCl, 10% glycerol), and protein concentrations were determined using the BCA Protein Assay kit (Pierce, Rockford, IL, USA). Protein lysates (10 *μ*g) were mixed with NuPage sample buffer and reducing agent (Invitrogen, Carlsbad, CA, USA), and then heated at 70°C for 10 min. Protein lysates were then separated on a 10% Tris–HCl polyacrylamide gel (BioRad, Hercules, CA, USA) using the Min-Protein 3 cell (BioRad) according to the manufacturer's protocol. Separated proteins were transferred to an Immuno-Blot PVDF membrane (BioRad) using the Mini Trans-Blot electrophoretic transfer cell and protocol (BioRad). Oestrogen receptor-*α* mouse monoclonal antibody (NCL-ER-6F11, Nova Castra, Newcastle, UK) was used at 1 : 100, and *β*-actin (Cell Signaling Technology) at 1 : 1000.

### Cell-cycle analysis

Cells were grown in culture medium containing charcoal/dextran-treated FBS (reduced serum) for 48 h for synchronisation and harvested near 80% confluence. Adherent cell cultures were harvested by trypsinisation, re-suspended in PBS, and fixed with 75% ethanol for 2 h at 4°C. Fixed cells were treated with 10 *μ*g ml^−1^ RNase A and DNA was stained with propidium iodine (100 *μ*g ml^−1^) for 30 min. Single cells (1 × 10^6^ cells ml^−1^) were analysed with the FACSCalibur and CellQuest Software (Becton Dickinson, Franklin Lakes, NJ, USA). Cell-cycle analysis was performed using ModFit LT 2.0 software (Verity Software House, Topsham, ME, USA).

### Statistics

The Mann–Whitney *U*-statistical test was used to determine the association of mRNA levels for selected genes with tumours expressing either basal CKs or ER. Student's two-tailed *t*-tests were used for planned comparisons between MCF-7 and TMX2-28 for each cell-cycle stage, and for comparison of mRNA levels between TMX2-28 and each of the four breast cell lines. Significance was set at *P*⩽0.05.

## RESULTS

### Characterisation of TMX2-28 cells

**Figure 1:**
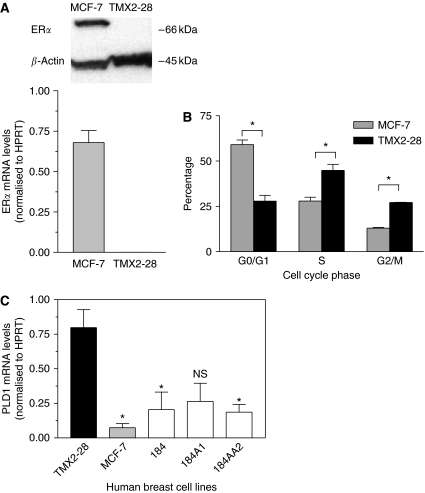
Phospholipase D1 (PLD1) is overexpressed in the aggressive, oestrogen receptor (ER)-negative variant of MCF-7 cells, TMX2-28. (**A**) Cell cultures were grown to subconfluence, and RNA and protein were purified. Oestrogen receptor-*α* mRNA expression was determined by real-time RT–PCR (bottom), and protein expression was determined by western immunoblotting (top). Experiments were repeated two times; the bottom panel shows the mean and s.d., and the top panel shows a representative image from one experiment. (**B**) Cell-cycle analysis was determined by propidium iodine staining and FACS analysis. The mean and s.d. from two experiments are shown. The per cent of TMX2-28 cells in each phase of the cell cycle differed significantly from the per cent of MCF-7 cells in the same phase (*P*<0.05). (**C**) Total RNA was isolated from cell cultures, and mRNA levels were determined by real-time RT–PCR. Data for each cell line are normalised to hypoxanthine ribosyltransferase (HPRT) and are represented as mean and s.d. from two biological samples each tested in duplicate. Levels of PLD1 mRNA were significantly higher in TMX2-28 cells than in MCF-7, 184, and 184AA2 cells (*P*<0.05). The same trend was apparent between TMX2-28 and 184A1 cells, but the means were not significantly different (*P*=0.056).

The TMX2-28 cell line was 1 of 28 clones derived from human MCF-7 breast cancer cells grown in the presence of the anti-oestrogen, tamoxifen, for 6 months (Fasco *et al*, 2003; Pentecost *et al*, 2005). As shown in Figure 1A, TMX2-28 cells do not express ER*α* mRNA or protein. Comparison between the cell-cycle progression of the ER-negative TMX2-28 cells and the ER-positive MCF-7 cells revealed increased mitotic activity of TMX2-28 cells (Figure 1B). Significantly more TMX2-28 cells were in S and G_2_/M phases (1.6 and 2.0 times greater) and fewer were in G_0_/G_1_ phase, as compared to MCF-7. These data suggest that TMX2-28 cells have lost G_0_/G_1_ regulation of the cell cycle, resulting in increased cell division and proliferation. Furthermore, TMX2-28 cells are more invasive than are MCF-7 cells (Gozgit *et al*, 2006), leading us to characterise TMX2-28 as an aggressive ER-negative breast cancer cell line.

Recent reports have shown that ER-negative tumours frequently express the basal-like gene signature and are correlated with poor clinical outcome (Perou *et al*, 2000; Sorlie *et al*, 2001; Abd El-Rehim *et al*, 2004). Since TMX2-28 cells have lost expression of the ER and show an aggressive phenotype, we examined the mRNA levels of the seven CKs listed in Table 1 to determine if TMX2-28 cells express a similar basal-like CK gene signature. We found that TMX2-28 cells display an altered CK mRNA expression profile compared to MCF-7 cells; this profile is similar to the mixed basal/luminal phenotype recently described for a subset of breast carcinomas (Abd El-Rehim *et al*, 2004). TMX2-28 cells express much higher levels of basal CK mRNAs (CKs 5, 14, and 17) than do MCF-7 cells (Table 1). Luminal CKs 8 and 18 are similarly expressed in TMX2-28 and MCF-7 cells; however, TMX2-28 cells have lost expression of luminal CKs 19 and 20. Although TMX2-28 cells overexpress CKs 5, 14, and 17 compared to MCF-7 cells it is important to note that TMX2-28 cells express much lower levels of these basal CKs than do the 184 HMECs, which display a basal phenotype.

To further examine the potential differences between TMX2-28 and MCF-7 cells, we compared the global gene expression of the two cell lines using cDNA microarray analysis. cDNA microarray comparison of TMX2-28 and MCF-7 cells resulted in 1402 differentially expressed transcripts (Supplementary Table 1). Two hundred transcripts were upregulated, and 1202 were downregulated in TMX2-28 compared to MCF-7 cells. Using NetAffx software (Liu *et al*, 2003), we sorted the 200 upregulated transcripts by biological function, with specific attention on genes involved in cell-cycle control and proliferation, and we found that PLD1 was expressed at a level 2.1-fold higher in TMX2-28 cells than in MCF-7 cells.

Using real-time RT–PCR, we found that PLD1 was overexpressed by 10-fold in TMX2-28 cells compared to MCF-7 (Figure 1C). We also evaluated the expression of PLD1 in the non-tumorigenic HMEC lines 184 (finite lifespan), 184A1 (immortalised), and 184AA2 (immortalised, p53 null). We found that while PLD1 was moderately expressed in these non-tumorigenic breast cell lines, it was expressed significantly more in TMX2-28 (Figure 1C).

### PLD1 mRNA expression in clinical breast tumours

**Figure 2:**
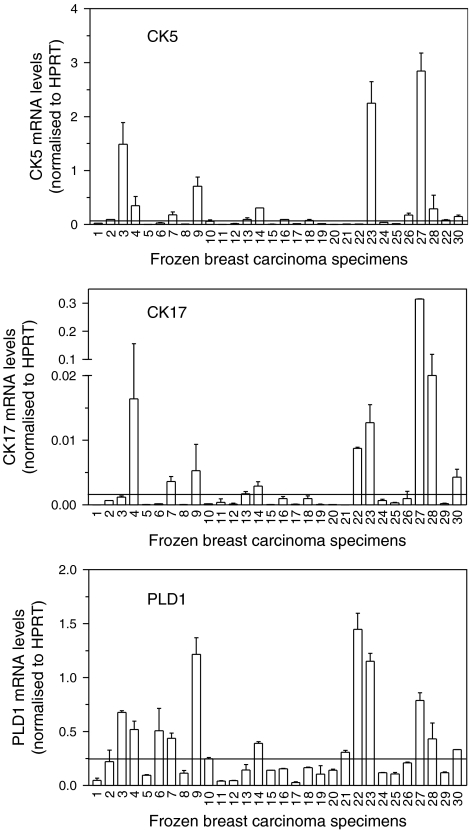
Phospholipase D1 (PLD1) mRNA levels are highly expressed in basal-like breast carcinomas. Total RNA was isolated from frozen human–breast tumour specimens, and gene expression was determined using real-time RT–PCR. An arbitrary cutoff point was used to select tumours that expressed high levels of cytokeratin (CK5), CK17, and PLD1. Phospholipase D1 mRNA levels were significantly higher in tumours that expressed high levels of CK5 and/or 17 (*P*<0.01). Phospholipase D1 mRNA levels were not significantly higher in oestrogen receptor (ER)-negative tumours (1–18 are ER positive, and 19–30 are ER negative, *P*>0.05, see text for details). Samples were tested two or three times; s.d. represents variation between PCR experiments.

To determine whether PLD1 gene expression correlates with either the basal or luminal CKs and/or ER status in breast tumour tissue, we isolated RNA from 30 frozen human–breast carcinomas and evaluated gene expression by real-time RT–PCR. We used an arbitrary cutoff point to score high-mRNA expression of CK5 (>0.1 arbitrary units) and CK17 (>0.002 arbitrary units). We identified 11 tumours that expressed high-mRNA levels for either CK5 and/or 17 (Figure 2). Of these 11 tumours, 8 expressed both CKs 5 and 17, while 2 expressed only CK5, and 1 expressed only CK17. Five of the 11 tumours that expressed either CK5 and/or 17 did not express luminal CKs 8/18, whereas the remaining six tumours expressed a mixed basal/luminal CK expression profile (data not shown). The levels of PLD1 mRNA were evaluated in the 30 frozen human–breast carcinomas. Phospholipase D1 mRNA levels were significantly greater (>0.25 arbitrary units) in tumours that scored high for CKs 5 and/or 17 expression (Figure 2; *P*<0.01). Next, we examined the relation between PLD1 expression and ER status. Oestrogen receptor status was obtained from the pathology reports that accompanied each tumour specimen. Tumour specimens 1–18 are ER positive, and tumour specimens 19–30 are ER negative. Phospholipase D1 mRNA expression was not correlated with ER status (*P*>0.05).

**Figure 3:**
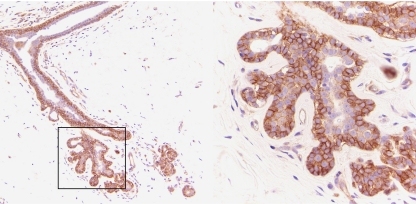
Phospholipase D1 (PLD1) expression in tissues from reduction mammoplasty. Phospholipase D1 shows moderate expression in tissue specimens from reduction mammoplasty as determined by immunohistochemistry (IHC); a representative image of PLD1 staining is shown. Phospholipase D1 immunoreactivity was concentrated in the outer basal/myoepithelial cell layer of the terminal ductal lobular units (TDLUs) of the breast. An enlarged image of cells with a TDLU showing PLD1 expression in the outer basal/myoepithelial cell layer is shown on the right.

We also evaluated the expression of PLD1 in tissues from reduction mammoplasty. We found moderate PLD1 protein expression in 4 of 10 tissues from reduction mammoplasty. An example of protein expression in a tissue specimen from reductive mammoplasty is shown in Figure 3. The other six tissues from reductive mammoplasty showed weak staining (baseline level expression) for PLD1. Interestingly, PLD1 protein was detected specifically in the outer basal/myoepithelial cell layer within the terminal ductal lobular units of the breast (see Figure 3), suggesting a potential role for PLD1 in the basal/myoepithelial cells, as opposed to the luminal cells.

### Immunohistochemical analysis of PLD1, phospho-Akt and phospho-mTOR in clinical breast carcinomas

**Figure 4:**
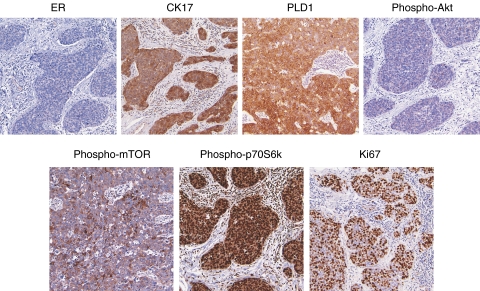
Protein-expression profile of tumour specimen no. 22. The matching formalin-fixed, paraffin-embedded (FFPE) tissue for tumour specimen no. 22 (see Figure 2) was sectioned, and protein expression was determined by immunohistochemistry (IHC). The staining profile for this tumour demonstrates that the phospholipase D1 (PLD1)/mammalian target of rapamycin (mTOR)/p70S6K pathway is active in cytokeratin (CK17)-positive tumour cells that are negative for phospho-Akt and ER expression.

Akt activity is frequently overexpressed in HER2-positive breast tumours (Tokunaga *et al*, 2006), and basal-like tumours tend to have low HER2 levels (Perou *et al*, 2000; Nielsen *et al*, 2004). We have found that PLD1 mRNA levels are significantly higher in basal-like tumours (Figure 2); therefore, we reasoned that PLD1 may be inversely expressed with phospho-Akt in breast tumours with basal-like features. We began by evaluating the expression profile of a breast tumour specimen that had high-PLD1 mRNA levels (shown in Figure 2). As shown in Figure 4, tumour specimen no. 22 is ER negative, expresses the basal marker CK17, and is PLD1 positive. Cancer cells in this tumour are negative for phospho-Akt, but show positive cytoplasmic expression for activated phospho-mTOR and positive nuclear phosphorylated expression for the downstream mTOR effector, p70S6k. This particular breast tumour represents a case in which PLD1 and phospho-Akt are inversely expressed, and the PLD1/mTOR/p70S6k pathway is active in the cancer cells. Figure 4 also illustrates that this tumour shows high expression for the proliferation marker, Ki67; the pathology report indicated that this is a high-grade tumour. This tumour represents a type that does not express targets for either anti-hormone or -HER2 therapies. Therefore, identification of new molecular pathways such as PLD1/PA/mTOR signalling that are constitutively active in such types of tumours may be useful in selecting alternative targeted therapies.

**Figure 5:**
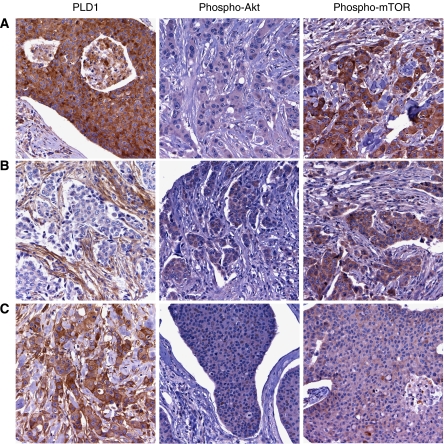
Phospholipase D1 (PLD1) and phospho-Akt are inversely expressed in a subset of breast carcinomas. Tissue microarrays (TMAs) were constructed with archived formalin-fixed, paraffin-embedded (FFPE) breast carcinomas, and protein expression was determined by immunohistochemistry (IHC). Representative photographs are shown: row (**A**), tumour cells show positive staining for PLD1, negative for phospho-Akt, but positive for phospho-mammalian target of rapamycin (mTOR); row (**B**), PLD1 shows positive expression in stromal cells, but is negative in the tumour cells, whereas phospho-Akt and -mTOR show positive expression in tumour cells; row (**C**), shows positive expression for PLD1, phospho-Akt, and phospho-mTOR.

To evaluate further the relationship of PLD1 with phospho-Akt, we used TMA and IHC technology to examine 42 breast carcinomas. The design of the TMA is described in the Materials and Methods section. Oestrogen receptor expression was determined from the pathology reports that accompanied each tumour specimen, and the expression of PLD1, phospho-Akt, phospho-mTOR, and CKs 5/17 was evaluated by IHC analysis (Table 2). Phospholipase D1 was expressed in 27 of the 42 breast tumours. Seventeen tumours had a basal level of PLD1 expression (score of 1), whereas the other 10 tumours overexpressed PLD1 (score of 2 or 3). Phospholipase D1 was overexpressed in 6 of the 28 ER-positive tumours and in 4 of the 11 ER-negative cases. Although only four tumours scored positive for CK5 and/or CK17 expression, it is interesting to note that PLD1 was overexpressed in 3 of the 4 CK5/17-positive cases, the basal-like CK-expression pattern. Of the 10 tumours that overexpressed PLD1, 5 were negative for phospho-Akt expression, and importantly, these 5 PLD1-positive tumours overexpressed phospho-mTOR (Table 2, tumours 6–10). Representative images of a tumour that overexpressed PLD1 and phospho-mTOR, but did not express phospho-Akt, are shown in Figure 5A. These data indicate that PLD1 may be activating the mTOR pathway in these tumours that do not have constitutive phospho-Akt expression. Seven tumours showed positive phospho-Akt and negative PLD1 expression, and five of these tumours scored positive for phospho-mTOR. Representative images of a tumour that stained negative for PLD1 and positive for both phospho-Akt and phospho-mTOR are shown in Figure 5B. Lastly, five tumour specimens overexpressed both PLD1 and phospho-Akt, and two of these five tumours overexpressed phospho-mTOR. An example of a tumour overexpressing all three proteins is shown in Figure 5C. Interestingly, 6 of the 10 tumours that overexpressed PLD1 are grade 3 tumours, and 5 of these tumours are among the 7 tumours that overexpress PLD1 along with phospho-mTOR. These data suggest the possibility that PLD1/mTOR signalling plays a role in the growth of high-grade tumours; and a larger study evaluating the expression of these proteins with tumour grade and patient prognosis is clearly warranted.

## DISCUSSION

Phospholipase D1 has been implicated in breast cancer tumorigenesis and has been proposed to play a role in oestrogen-independent growth (Foster and Xu, 2003; Rodrik *et al*, 2005). Two previous reports examined PLD activity and expression, both in a relatively small number of breast cancers. Elevated PLD activity was found in 17 out of 19 microsomal preparations from human breast tumours as compared to microsomal preparations from adjacent normal breast tissue (Uchida *et al*, 1997) and PLD mRNA and protein levels were overexpressed in 14 of 17 breast cancer tissues compared to normal (Noh *et al*, 2000). Our findings that PLD1 is overexpressed in TMX2-28 breast cancer cells and in a subset of breast tumours are consistent with the published data.

**Figure 6:**
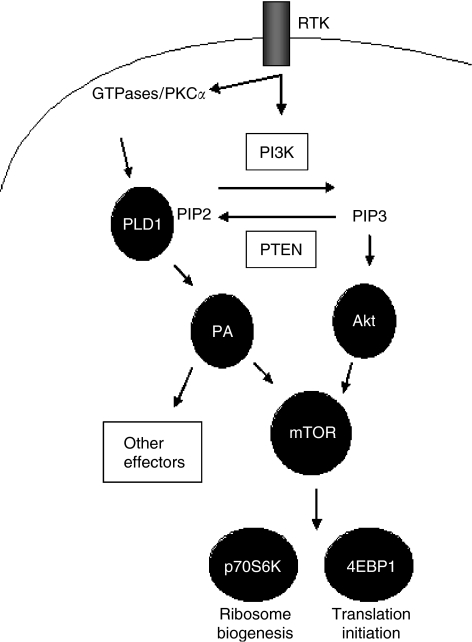
Model of alternative mammalian target of rapamycin (mTOR) Activation by PLD1 in breast cancer. Receptor tyrosine kinases (RTKs) activate several downstream signals including small GTPases, PKC*α*, and PI3K. Phospholipase D1 (PLD1) is activated, by either members of the small GTPase family or by PKC*α*, and is dependent on phosphatidylinositol-4,5-bis-phosphate (PIP2) to generate phosphatidic acid (PA), whereas Akt requires PIP3 for localisation to the plasma membrane and activation. Phosphatidic acid or phosphorylated Akt activates the protein kinase mTOR, which phosphorylates its downstream effectors p70S6K and 4E-BP1, thereby promoting cell growth and proliferation (adapted from Foster and Xu, 2003).

In this study, we are the first to demonstrate that the PLD1/mTOR pathway is active in a subset of clinical breast carcinomas that are negative for phospho-Akt. This finding is consistent with results reported by Chen *et al* (2005) who showed that PLD1 and phospho-Akt are inversely expressed in a subset of breast cancer cell lines. Taken together, their study and ours suggest that an alternative mTOR activation pathway is active in a subset of breast tumours (see Figure 6 for model). Patients with tumours expressing activated PLD1/mTOR signalling may be sensitive to rapamycin-based therapies; however, it has been shown that high levels of PLD1 confers rapamycin resistance in MDA-MB-231 breast cancer cells *in vitro* (Chen *et al*, 2003). Although this is an important finding it is limited to cell lines and clinical trials utilising phospho-Akt as well as PLD1 expression are needed to understand the sensitivity of rapamycin-based therapies. We also found that PLD1 and phospho-Akt are coexpressed in some breast tumours, a fact which could complicate the utility of rapamycin-based therapies, as patients expressing both proteins could differ in sensitivity to mTOR inhibitors. We found that PLD1 was expressed in six ER-positive tumours; four of these tumours showed positive phospho-mTOR expression, and two were negative for phospho-Akt expression. Therefore, it is plausible that those patients with tumours positive for ER and PLD1 will respond to combined anti-hormone and rapamycin-based therapies; that combination has been successful in inhibiting the proliferation of breast cancer cell lines (Boulay *et al*, 2005). Our data also show that most basal-like tumours express PLD1, and it has been shown that patients with these types of tumours suffer from poor prognosis (Sorlie *et al*, 2001; Abd El-Rehim *et al*, 2004). Although we limited our studies to the evaluation of PLD1 mRNA and protein it is important to point out that PLD1 expression may not always correlate with PLD1 activity. Therefore, it is plausible that tumour cells with basal PLD1 mRNA/protein may still have altered PLD1 enzyme activity. Furthermore, a second isoform of PLD, PLD2, may play a role in the transformed phenotype (Foster and Xu, 2003); however, in our study we limited the analysis to PLD1.

Rapamycin-based therapies target mTOR and are in clinical trials for various cancers including breast (Carraway and Hidalgo, 2004). Studies on breast cancer cell lines have revealed that the rapamycin analogue CCI-779 was most effective in inhibiting growth in cell lines that were ER-dependent, that overexpressed HER-2 and/or had PTEN deletions, and that expressed high levels of activated Akt (Carraway and Hidalgo, 2004). Others have suggested that patients with breast tumours displaying aberrant PI3K/Akt/mTOR signalling will benefit the most from rapamycin-based therapies, because tumour cells that have abnormal expression in the PI3K/Akt/mTOR pathway may depend on this pathway for growth, and therefore may be sensitive to mTOR inhibitors (Hidalgo and Rowinsky, 2000; Carraway and Hidalgo, 2004; Thomas, 2006). Data from our study identify an additional subset of patients with tumours that overexpress PLD1 and phospho-mTOR, but do not express constitutive levels of phospho-Akt.

In this report, we have used the MCF-7-derived cell line, TMX2-28, as a model to study ER-negative breast cancer. Gene-expression studies identified the overexpression of PLD1 in TMX2-28 cells. We are the first to report that PLD1 is highly expressed in some phospho-Akt-negative clinical breast tumours. These data support the studies by Chen and colleagues that suggest the presence of an alternative mTOR activation pathway in breast cancer cell lines with high-PLD1 activity and relatively low-phospho-Akt levels. Phospholipase D1 tends to be overexpressed in tumours that express high levels of CKs 5/17, markers of basal-like tumours, and others have shown basal-like tumours are frequently associated with poor prognosis. On the basis of these studies we speculate that PLD1 may serve as a biomarker for selecting patients for rapamycin-based therapies; a larger follow-up study to evaluate the clinical utility of PLD1 is clearly warranted.

## Figures and Tables

**Table 1:** Comparison of cytokeratin mRNA expression profile among TMX2-28, MCF-7, and 184 HMEC cells

	**Basal cytokeratins**			
**Cell line**	**5**	**14**	**17**	
TMX2-28	5	1100	0.67	
MCF-7	0.007	4.6	0.019	
184 HMEC	254	52991	689	
				

	**Luminal cytokeratins**			
	**8**	**18**	**19**	**20**
TMX2-28	633	1050	78	0.011
MCF-7	956	856	967	939
184 HMEC	30	54	<0.0001	<0.0001

Relative mRNA levels determined by real-time RT–PCR normalised to HPRT.

**Table 2:** Immunohistochemical analysis of PLD1 in human breast carcinomas

**Tumour category**	**Tumour ID**	**PLD1**	**Phospho-Akt**	**Phospho-mTOR**	**CK5**	**CK17**	**ER**	**Grade**
PLD1 and phospho-Akt overexpressed	1	3	2	1	0	0	Pos	II
	2	2	2	1	0	3	Neg	III
	3	2	2	3	0	0	Pos	III
	4	2	2	2	0	3	Pos	III
	5	2	2	1	0	0	Pos	I
								
PLD1 overexpressed and phospho-Akt low or negative	6	3	1	3	0	0	Neg	III
	7	2	1	2	0	0	Pos	II
	8	2	0	2	3	0	Neg	III
	9	2	0	2	0	0	Pos	III
	10	2	0	2	0	0	Neg	I
								
PLD1 positive (low)	11	1	2	3	0	0	Pos	II
	12	1	2	3	0	0	Pos	II
	13	1	2	2	0	0	Pos	I
	14	1	2	1	0	0	Pos	II
	15	1	1	2	0	0	Unk	I
	16	1	1	2	0	0	Pos	II
	17	1	1	1	0	0	Unk	I
	18	1	1	0	0	0	Pos	III
	19	1	1	3	0	0	Unk	II
	20	1	1	1	0	0	Pos	II
	21	1	0	0	0	0	Pos	III
	22	1	0	3	0	0	Pos	III
	23	1	0	1	0	0	Neg	III
	24	1	0	3	0	0	Neg	II
	25	1	0	3	0	0	Pos	III
	26	1	0	3	0	0	Pos	II
	27	1	ND	ND	0	0	Neg	Unk
								
PLD1 negative	28	0	2	2	0	0	Pos	II
	29	0	2	0	0	0	Pos	II
	30	0	2	2	0	0	Neg	III
	31	0	1	3	3	3	Neg	III
	32	0	1	1	0	0	Pos	III
	33	0	1	2	0	0	Pos	II
	34	0	0	2	0	0	Pos	II
	35	0	0	2	0	0	Pos	I
	36	0	0	2	0	0	Pos	I
	37	0	0	2	0	0	Neg	II
	38	0	0	0	0	0	Pos	III
	39	0	0	0	0	0	Neg	III
	40	0	0	0	0	0	Pos	III
	41	0	0	0	0	0	Pos	II
	42	0	0	0	0	0	Pos	II
								
PLD1 not determined	43	ND	0	1	0	0	Pos	I
	44	ND	ND	ND	0	0	Neg	III
	45	ND	1	3	0	0	Pos	II
	46	ND	1	0	0	0	Pos	II

ND=expression not detected due to inadequate tissue represented on TMA; Unk=ER status unknown.

IHC was scored on a scale of 0–3, where 0=no staining, 1=weak or basal level, 2=moderate, and 3=strong. A score of moderate or strong expression is considered overexpressed.
